# ‘Sepsis-related anemia’ is absent at hospital presentation; a retrospective cohort analysis

**DOI:** 10.1186/s12871-015-0035-7

**Published:** 2015-04-24

**Authors:** Geertje Jansma, Fellery de Lange, W Peter Kingma, Namkje AR Vellinga, Matty Koopmans, Michael A Kuiper, E Christiaan Boerma

**Affiliations:** Department of Intensive Care, Medical Center Leeuwarden, Leeuwarden, the Netherlands

**Keywords:** Sepsis, Hemoglobin concentration, Anemia, Glycocalyx, Fluid balance

## Abstract

**Background:**

Anemia is a common feature during sepsis that occurs due to iatrogenic blood loss, depression of serum iron levels and erythropoietin production, and a decreased lifespan of erythrocytes. However, these mechanisms are unlikely to play a role in anemia at the start of sepsis. Moreover, sequestration of fluids, renal failure and increase of intravascular space may additionally influence the change in hemoglobin concentration during intravenous fluid administration in the acute phase of sepsis.

**Methods:**

In this retrospective study, patients who were admitted acutely to the Intensive Care Unit (ICU) were included. Patients who fulfilled the international criteria for severe sepsis or septic shock were included in the sepsis group (S-group). The remaining patients were allocated to the control group (C-group). Laboratory data from blood samples taken at first presentation to the hospital and at admission to the ICU, the amount of intravenous fluid administration and length of stay in the emergency department were collected and tested for significant differences between groups.

**Results:**

The difference in hemoglobin concentration between the S-group (n = 296) and C-group (n = 320) at first presentation in hospital was not significant (8.8 ± 1.2 versus 8.9 ± 1.2 mmol/l, respectively, p = 0.07). The reduction in hemoglobin concentration from the first presentation at the emergency department to ICU admission was significantly greater in the S-group compared to the C-group (1 [0.5-1.7] versus 0.5 [0.1-1.1] mmol/l, (p < 0.001)). Spearman rho correlation coefficients between the reduction in hemoglobin concentration and the amount of intravenous fluids administered or the creatinine level in the emergency department were significant (0.3 and 0.4, respectively, p < 0.001). In a multivariate regression analysis, creatinine, the amount of fluid administration and the presence of sepsis remained independently associated.

**Conclusions:**

Prior to in-hospital intravenous fluid administration, there is no significant difference in hemoglobin concentration between acute septic patients and acutely ill controls. Within several hours after hospital admission, there is a significant reduction in hemoglobin concentration, not only associated with the amount of intravenous fluids administered and the creatinine level, but also independently with sepsis itself.

## Background

In Europe, the incidence of sepsis in patients admitted to an Intensive Care Unit (ICU) is 37%, with a mortality rate of 27% during the ICU stay [1]. A common feature during sepsis is the development of anemia. Of note, the hematocrit of patients with sepsis in the ICU setting has been reported to be significantly lower in comparison with patients in the emergency department (ED) [2]. Previous studies have suggested multiple causes including iatrogenic blood loss, depression of serum iron levels and erythropoietin production, and a decrease in the lifespan of erythrocytes [3-9]. However, these studies have all been performed after considerable medical intervention. As the lifespan of erythrocytes is 120 days, and the production of new erythrocytes after activation of the erythropoiesis lasts several days [10], these factors will not cause anemia in the acute phase of sepsis. Theoretically, it is conceivable that in the acute phase of sepsis several other potential mechanisms may influence the hemoglobin (Hb) concentration. On the one hand, endothelial activation may lead to increased vascular permeability and fluid sequestration to the interstitium, leading to hemoconcentration [11,12]. On the other hand, degradation of the glycocalyx has been reported [13-15]. Shedding of this carbohydrate-rich layer coating the endothelium may not only lead to a substantial increase of intravascular space, but also to a release of previously encapsulated fluids into the vascular space, and thus may cause hemodilution. However, the previously described mechanisms are also related to fluid administration during this process. We hypothesized that during the first hours of hospital admission, intravenous fluid administration has a greater effect on the Hb concentration then these potential mechanisms and may be the leading cause for a reduction in Hb concentration. The aim of this study was to determine the incidence of anemia in the acute phase of sepsis at hospital admission, prior to intravenous fluid administration. Furthermore, we aimed to describe the relationship between the reduction in Hb concentration and intravenous fluid administration.

## Methods

### Patients and setting

In this retrospective, single-center cohort study, a general hospital database was used with the clinical information of all patients admitted to a closed-format 22-bed, mixed ICU in a tertiary hospital. Patients admitted for non-elective ICU indications were selected from this database. Based upon available medical correspondence and clinical information concerning the reason for admission, patients were identified as the sepsis-group (S-group) or the control-group (C-group). The S-group consisted of patients with severe sepsis and septic shock, according to the international criteria for sepsis [16]. The remaining patients formed the C-group. Exclusion criteria, based upon potential confounders for Hb concentration, are summarized in Table 1. We obtained ethical approval for our study from the local ethical committee (Regionale Toetsingscommissie Mensgebonden Onderzoek, Leeuwarden, the Netherlands). Due to the retrospective design of the study, the need for informed consent was waived.

**Table 1:** **Exclusion criteria**

General:	Factors of potential influence on the Hb concentration:
<18 years of age	- Pregnancy
No laboratory test results available	- High-Energetic Trauma or multiple trauma
Transfer of patient from other hospitals	- (Active) bleeding or documented blood loss 6 weeks prior to ICU admission
Unclear diagnosis	- Surgery 6 weeks prior to ICU admission
Admission for observation during treatment with thrombolytics	- Pre-existent untreated or refractory anemia
	- Chronic renal dysfunction with creatinine > 177 micromol/l or hemodialysis
	- Hematological or metastasized malignancy
	- Treatment with bone marrow suppressive medication (for example chemotherapy)
	- Admission after cardiac arrest
	- Transfer to ICU because of complications that occurred during elective hospital admission

### Data collection

Data on the general characteristics of patients were collected, including the severity of illness scores, according to the Acute Physiology and Chronic Health Evaluation (APACHE) II score, and the existence of co-morbidities, listed in Table 2. In all of the patients, blood samples were taken immediately after presentation to the ED and prior to in-hospital intravenous fluid administration. The primary outcome was Hb concentration at presentation to the ED. In a subgroup of patients, directly transferred from the ED to the ICU without prior transfer to a general hospital ward, measurements of Hb concentration were performed within one hour after ICU admission. The amount of intravenous fluids administration and lengths of stay in the ED were collected from patient records. The secondary outcome was the change in Hb concentration between the ED and ICU admission, and the correlation with the amount of intravenous fluid administration.

**Table 2:** **Baseline characteristics**

Variable	S-group (n = 296)	C-group (n = 320)	P-value
**Age (years)**	64 [50–76]	57 [41–70]	<0.001
**Sex (man, %)**	167 (56.4%)	180 (56.3%)	0.97
**APACHE-II score**	22 [16–27]	19 [12-24]	<0.001
**Diagnosis category**			
***Respiratory***		58 (18.1%)	
***Neurologic***		56 (17.5%)	
***Endocrine***		26 (8.1%)	
***Emergency surgery***		18 (5.6%)	
***Auto-intoxication***		77 (24.1%)	
***Cardiac***		55 (17.2%)	
***Other***		30 (9.4%)	
**Comorbidities**			
***Diabetes mellitus***	60 (20.3%)	57 (17.8%)	0.44
***Hypertension***	70 (23.6%)	66 (20.6%)	0.37
***Hypercholesterolemia***	19 (6.4%)	23 (7.2%)	0.71
***COPD***	32 (10.8%)	38 (11.9%)	0.68
***Alcohol abuse***	35 (11.8%)	25 (7.8%)	0.09
***Carcinoma***	7 (2.4%)	7 (2.2%)	0.88

Data are presented as median [IQR] or absolute number (%). COPD: Chronic Obstructive Pulmonary Disease. APACHE: Acute Physiology and Chronic Health Evaluation.

### Statistical analysis

The Statistical Package for the Social Sciences (SPSS for Windows; SPSS Inc., Chicago, IL, USA) was used for the statistical analysis. For continuous variables, all data are presented as mean ± SD, or as medians with interquartile ranges [IQR] in the case of non-normal distribution. Comparison between groups was performed by the Student’s *t*-test in the case of normal distribution and the Mann–Whitney *U* test in the case of non-normal distribution. Categorical variables are presented as frequencies (%) and were compared by the Chi-Square test. To assess correlations between variables with non-normal distributions a spearman rho correlation test was performed. All differences in variables between groups with a p value <0.25 in the univariate regression analysis were included in a multivariate regression analysis. To obtain a homogeneous dispersion in the residuals, a transformation had to be applied to a few non-normally distributed variables. A two-sided *p* value <0.05 was considered to be statistically significant.

## Results

Out of 2,500 non-elective ICU admissions during the period of May, 2006 until February, 2012, 296 patients could be identified in the S-group and 320 patients in the C-group. Patients in the S-group were older and had higher APACHE-II scores than patients in the C-group (Table 2).

### Primary outcome

At first presentation to the ED, there was no significant difference in the Hb concentration between both groups (Table 3). In males, the percentage of patients with anemia (Hb concentration below the lower reference value 8.5 mmol/l) was 26% in the S-group and 24% in the C-group (p = 0.68). For females, these percentages were 23% versus 12%, respectively (p = 0.02). Hemoconcentration (Hb concentration above the upper reference value) in males was present in 2% in the S-group and 3% in the C-group (p = 0.73). For females, this percentage was 5% in both groups (p = 0.46).

**Table 3:** **Laboratory measurements at first presentation in hospital**

Variable	S-group (n = 296)	C-group (n = 320)	P-value
**Hemoglobin (mmol/l)**	8.8 ± 1.2	8.9 ± 1.2	0.07
***Male***	9.1 ± 1.1	9.3 ± 1.1	0.14
***Female***	8.3 ± 1.1	8.5 ± 1.1	0.2
**Hematocrit (l/l)**	0.43 [0.38-0.46]	0.43 [0.39-0.47]	0.15
***Male***	0.44 [0.40-0.48]	0.45 ± 0.05	0.19
***Female***	0.40 [0.36-0.44]	0.40 [0.37-0.44]	0.24
**Mean corpuscular volume (fl)**	91 [87–95]	91 [87–94]	0.87
**C-reactive protein (mg/l)**	159 [35.5-303.5]	7 [3–27.5]	<0.001
**Leukocytes (x10^9/l)**	13.4 [9.4-18.4]	12.2 [9.2-16.7]	0.07
**Thrombocytes (x10^9/l)**	249 [178–346]	275 [226–349]	0.002
**Creatinine (μmol/l)**	108 [77–162]	84 [68–113]	<0.001
**Urea (mmol/l)**	8.4 [5.9-14.4]	6.0 [4.3-8.5]	<0.001
**Lactate dehydrogenase (U/l)**	241 [191 – 356]	226 [176–290]	0.006
**Bilirubin total (μmol/l)**	13 [9-22]	8 [5-11]	<0.001

Data are presented as mean ± SD or as median [IQR]. S-group: sepsis group. C-group: control group. Reference values hemoglobin concentration male 8.5-11 mmol/l, female 7.5-10 mmol/l; Hematocrit male 0.41-0.51 (l/l), female 0.36-0.47 (l/l).

Creatinine and urea levels, markers for possible chronic renal-failure induced anemia, were mildly but significantly higher in the S-group in comparison to the C-group. Furthermore, lactate dehydrogenase (LDH) and bilirubin levels, markers for possible hemolysis, were significantly higher in the S-group in comparison to the C-group. Spearman rho correlation coefficients between Hb concentration and LDH or bilirubin were 0.14 and 0.14, respectively (p = 0.001). After correction for potential confounders by multivariate linear regression analyses, the Hb concentration in the S-group was 0.23 mmol/l lower in comparison to the C-group (p = 0.01, R^2^ = .16; Table 4).

**Table 4:** **Multivariate linear regression analysis with hemoglobin concentration at presentation to the emergency department as dependent variable**

Independent variable	Beta coefficient	Standard error	P-value
**1/LDH (U/l)**	−97.58	0.19	<0.001
**Sex**	−0.83	24.06	<0.001
**Inclusion group**	−0.23	0.09	0.011

Model adjusted R^2^ = 0.16, F = 36.86, p < 0.001. Independent variables included in the stepwise multivariate linear regression analysis were APACHE-II score, 1/creatinine, 1/LDH, inclusion group and sex. To obtain a homogeneous dispersion in the residuals, a transformation had to be applied to the independent variables creatinine and LDH. For the variable sex: 0 = man and 1 = female. For the variable inclusion group: 1 = C-group and 2 = S-group.

### Secondary outcome

**Figure 1:**
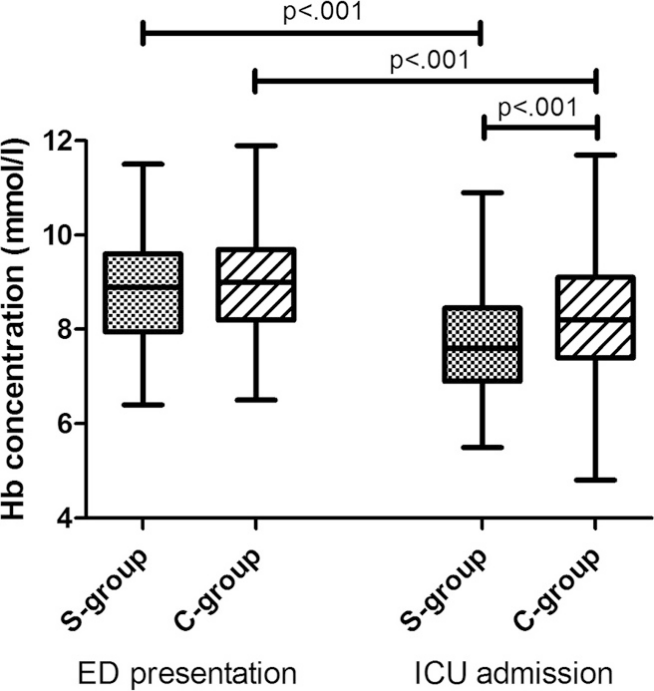
Hemoglobin concentration over time. Boxplots of the hemoglobin (Hb) concentration at presentation at the emergency department (ED) and at admission to the intensive care unit (ICU). In both groups the Hb concentration is significantly lower at ICU admission compared to at ED presentation (p < 0.001). At ICU admission the Hb concentration is lower in the S-group compared to the C-group (p < 0.001).

Eighty-five patients in the S-group (29%) and 146 patients in the C-group (45%) were directly admitted to the ICU after first presentation to the ED, with available Hb concentration levels within one hour of ICU admission. In both groups the Hb concentration decreased significantly at ICU admission in comparison to the Hb at ED presentation (7.7 ± 1.1 versus 8.8 ± 1.1 mmol/l (p < 0.001) in the S-group and 8.3 ± 1.2 versus 9.0 ± 1.0 mmol/l (p < 0.001) in the C-group)(Figure 1). However, the drop in Hb concentration from ED presentation compared to ICU admission was greater in the S-group compared to the C-group (1 [0.5-1.7] versus 0.5 [0.1-1.1] mmol/l, p < 0.001).

Intravenous fluid administration during the stay in the ED was significantly different between both groups (p = 0.02). In the S-group, the amount of intravenous fluid administration was 1.5 [1–2.5] liters and in the C-group, 1 [0.5-2] liter. Lengths of stay in the ED was significantly longer in the S-group compared to the C-group (103 [72–175] minutes versus 83 [58–123] minutes, p = 0.002). The Spearman rho correlation coefficients between the reduction in Hb concentration and the amount of intravenous fluid administration or creatinine was 0.3 (p < 0.001) and 0.4 (p < 0.001), respectively. In a multivariate regression analysis, creatinine, the amount of fluid administration and the presence of sepsis remained independently associated (Table 5).

**Table 5:** **Multivariate linear regression analysis with hemoglobin concentration at intensive care unit admission as dependent variable**

Independent variable	Beta coefficient	Standard error	P-value
**Hemoglobin at ED (mmol/l)**	0.77	0.05	<0.001
**Amount of intravenous fluid**	−0.15	0.05	0.003
**administration at ED (l)**			
**Creatinine (μmol/l)**	−0.001	0.00	<0.001
**Inclusion group**	−0.39	0.11	0.011

Model adjusted R^2^ = 0.64, F = 71,17, p < 0.001. For the variable inclusion group: 1 = C-group and 2 = S-group.

## Discussion

In this study, we did not observe a significant difference in the Hb concentration between sepsis patients and controls, prior to in-hospital intravenous fluid administration. In males, the incidence of anemia was considerable, but did not differ between groups (26% in sepsis versus 24% in controls). In females however, the percentage of patients with anemia was higher in the sepsis patients compared to the controls (23% versus 12%). Absolute hemoconcentration was present in a small percentage of patients (≤5%) with no difference between groups. Within several hours after admission, the drop in Hb concentration was considerable and significant in both groups. Our observation that the reduction in Hb concentration in response to intravenous fluid administration was more pronounced in sepsis patients compared to controls (1 [0.5-1.7] versus 0.5 [0.1-1.1] mmol/l) is of note. In a multivariate analysis, this reduction was significantly associated with the amount of intravenous fluid administration, renal failure and sepsis itself. This drop in Hb concentration after intravenous fluid administration can be explained by three potential mechanisms that may take place separately or simultaneously. First, there may have been a significantly more profound hemoconcentration in the sepsis group in comparison to the non-sepsis group at baseline [17,18]. In this case, fluid administration ‘corrects’ a seemingly normal Hb concentration at baseline, thus revealing an absolute deficit in red blood cell volume. Second, the significantly higher creatinine in the sepsis group may reflect renal failure and the inability of the body to correct for hypervolemia with enhanced urine production [19]. Finally, sepsis itself may cause an increase in intravascular space. This increase in intravascular space may be due to the loss of vasomotor tone [20]. In this condition, intravenous fluid administration is necessary to counteract a reduction in cardiac output, thus contributing to the typical hyperkinetic state, with a consequent reduction in Hb concentration. In addition, shedding of the glycocalyx during sepsis occurs [13-15]. The estimated plasma volume fixed within this carbohydrate-rich layer is 700–1500 ml [21,22], and it is not part of the intravascular volume under non-pathologic conditions [23]. It is conceivable that the observed shedding of glycocalyx not only releases this volume into the intravascular circulation, but also increases the inner diameter of the microvascular circulation. The fact that the creatinine level, the amount of intravenous fluid administration and the presence of sepsis were all independently associated with the change in Hb concentration during intravenous fluid administration suggests this multi-causality. Our observations seem to be of interest for the clinical setting because one of the potential reasons for fluid administration in sepsis is the idea that increased vascular permeability leads to fluid sequestration into the interstitial compartment, and thus, should lead to hemoconcentration [18,24]. However, our data show that the net-result on Hb concentration is overshadowed by the above described potential mechanisms.

The study by Ba et al. demonstrates that Hb concentrations typically declines in all ICU patients during the first days of ICU stay with a greater decline in patients with sepsis, and continues to decrease beyond the third day in septic patients [9]. In this study the Hb concentration at ICU admission in septic patients was 7.4 ± 1.3 mmol/l (n = 28) and in non-septic patients 7.7 ± 1.3 mmol/l (n = 56). This study was not restricted to patients who were admitted to the ICU immediately after first presentation and treatment at ED, but also included patients after surgery. In the study by van Eijk et al., 92 patients with sepsis were enrolled after presentation to the ED and subsequent hospital admission [8]. With a median of 7.5 mmol/l, the Hb concentration was lower in comparison to our sepsis patients. However, in this study, blood samples were obtained in the later phase of sepsis, i.e., the first 14 days of hospital admission. In a small sample study by Piagnerelli et al., the Hb concentration within the first 24 hours of ICU admission was 6.9 ± 1.4 mmol/l in patients with sepsis [25]. The difference between our data and the above mentioned studies highlights the importance of the timeline. In our study, the short timeframe in which the Hb concentration reduced considerably and the clear correlation with intravenous fluid administration, both point towards an iatrogenic component in the development of what is referred to as “sepsis-related anemia”. Other potential mechanisms, including changes in iron metabolism and a shortened life span of erythrocytes, are unlikely to play a role within this short timeline. Furthermore, the positive correlation coefficients between bilirubin/LDH and Hb concentration are not in line with hemolysis, but rather a marker of organ dysfunction. This phenomenon is in line with previous reports [26]. The amount of blood taken for laboratory investigation was minimal.

Limitations of the present study are largely related to the design of the study. This is a retrospective study. Forty-two patients (1,7%) could not be identified in one of both groups as a result of incomplete documentation. In 62 patients out of 231 patients directly admitted to the ICU after first presentation to the hospital, the total amount of intravenous fluid administration was not recorded in the clinical data set. Although we did our best to identify all factors of potential influence on Hb concentration, we were unable to exclude the effect of intravenous fluid administration prior to first presentation in hospital. In the analysis of secondary outcomes, more patients in the C-group were admitted directly to the ICU after first presentation to the ED in comparison to the S-group, causing a potential bias.

## Conclusion

A common feature during sepsis is the development of anemia that seems to be caused by iatrogenic blood loss, depression of serum iron levels and erythropoietin production, and a decreased lifespan of erythrocytes. Our study demonstrates that during the acute phase of sepsis, prior to in-hospital intravenous fluid administration, the Hb concentration does not differ significantly from acutely ill controls. However, within several hours after hospital admission, there is a significant reduction in Hb concentration. This reduction is not only associated with the amount of intravenous fluids administered and the creatinine level, but it is also independently associated with sepsis itself.

## Abbreviations

ICU: Intensive Care Unit
Hb: hemoglobin
S-group: sepsis-group
C-group: control-group
INR: International Normalized Ratio
ED: Emergency Department
APACHE: Acute Physiology and Chronic Health Evaluation
COPD: Chronic Obstructive Pulmonary Disease
SD: Standard deviation
IQR: Interquartile ranges
LDH: Lactate dehydrogenase
